# Supplementary material for: Prevalence of bacteriologically-confirmed pulmonary tuberculosis in urban Blantyre, Malawi 2019–20: Substantial decline compared to 2013–14 national survey
Source: PLOS Glob Public Health. 2023 Oct 20;3(10):e0001911. doi: 10.1371/journal.pgph.0001911 (PMC10588852; doi:10.1371/journal.pgph.0001911)
Supplement: S1 Checklist — (DOCX) [file pgph.0001911.s001.docx]

STROBE Statement—checklist of items that should be included in reports of observational studies

|  | Item No. | Recommendation | Page  No. | Relevant text from manuscript |
| --- | --- | --- | --- | --- |
| **Title and abstract** | 1 | (*a*) Indicate the study’s design with a commonly used term in the title or the abstract |  |  |
|  |  | (*b*) Provide in the abstract an informative and balanced summary of what was done and what was found | 2 | “We conducted a TB prevalence survey” |
| Introduction | | | |  |
| Background/rationale | 2 | Explain the scientific background and rationale for the investigation being reported | 4-5 | “TB prevalence surveys … likely provide the least biased approach to estimating disease burden” |
| Objectives | 3 | State specific objectives, including any prespecified hypotheses | 5 | “The aim was to estimate the burden of TB amongst adults 18 years or older in middle-to-high density urban Blantyre, Malawi.” |
| Methods | | | |  |
| Study design | 4 | Present key elements of study design early in the paper | 6 | “a cluster-based, cross-sectional TB prevalence survey” |
| Setting | 5 | Describe the setting, locations, and relevant dates, including periods of recruitment, exposure, follow-up, and data collection | 6 | “between May 2019 and March 2020. Blantyre City is located in the Southern Region of Malawi, and has a population of approximately 800,250 mostly living in several informal urban settlements built on underserviced land” |
| Participants | 6 | (*a*) *Cohort study*—Give the eligibility criteria, and the sources and methods of selection of participants. Describe methods of follow-up  *Case-control study*—Give the eligibility criteria, and the sources and methods of case ascertainment and control selection. Give the rationale for the choice of cases and controls  *Cross-sectional study*—Give the eligibility criteria, and the sources and methods of selection of participants | 6-7 | “In each cluster, 115 households were randomly selected from a sampling frame of all household GPS co-ordinates obtained from Google Earth, aiming to recruit 215 adults (aged 18 and above) per cluster…. Household residents were defined as those who usually ate and slept in the same residence. All adult (18 years and over) household residents were eligible for participation if willing and able to provide written or witnessed informed consent.” |
|  |  | (*b*) *Cohort study*—For matched studies, give matching criteria and number of exposed and unexposed  *Case-control study*—For matched studies, give matching criteria and the number of controls per case | N/A | N/A |
| Variables | 7 | Clearly define all outcomes, exposures, predictors, potential confounders, and effect modifiers. Give diagnostic criteria, if applicable | 7-8 | “Participants with positive microscopy, Xpert or MTB culture results were classified as having bacteriologically-confirmed TB. For this survey, an Xpert MTB/Rif G4 trace result was considered positive for MTB. Smear-positive TB participants were defined as those with a direct smear indicating acid fast bacilli.” |
| Data sources/ measurement | 8* | For each variable of interest, give sources of data and details of methods of assessment (measurement). Describe comparability of assessment methods if there is more than one group | 7-8 | “A household questionnaire was conducted with one consenting adult household member (the household head if present) to capture household-level variables including socioeconomic indicators and the age and sex of all household residents. Individual questionnaires were then conducted with all consenting household members, including socio-demographics, a symptom screen for cough of any duration, and brief details of previous HIV and TB testing and care…. Chest X-ray used Min X-ray Commander CMDR-2S-T, with films classed as normal or having any abnormality by a trained radiographer, with reference to results of Qure.ai (version 2) computer-aided detection software…. HIV testing using OraQuick (OraSure) and Determine (Alere) finger-prick tests in parallel, with confirmation by Uni-Gold (Trinity Biotech) for positive results, was offered to all participants not on ART.” |
| Bias | 9 | Describe any efforts to address potential sources of bias | 6 | “115 households were randomly selected” |
| Study size | 10 | Explain how the study size was arrived at | 8-9 | “The calculated sample size of 14,511 participants was based on the ability to estimate an overall TB prevalence of 900 per 100,000 with absolute precision of +/- 250,000 per 100,000 (relative precision 27.8%) and a design effect to account for clustering of 2.25. Based on previous work by NTP and our research group in Blantyre, a relatively high non-participation rate of 25% was also assumed. This final sample was rounded up to 15,500 adults (215 per cluster). “ |

Continued on next page

| Quantitative variables | 11 | Explain how quantitative variables were handled in the analyses. If applicable, describe which groupings were chosen and why | 9 | “Data was summarised by frequencies, percentages, and medians as appropriate...” |
| --- | --- | --- | --- | --- |
| Statistical methods | 12 | (*a*) Describe all statistical methods, including those used to control for confounding | 8-10 | “Following WHO-recommended best-practice analytical methods, we estimated TB prevalence using logistic regression models with robust standard errors (calculated from observed between-cluster variability) to account for clustering…” |
|  |  | (*b*) Describe any methods used to examine subgroups and interactions | 9 | “chi-squared tests to examine differences between groups, such as participation rate by sex” |
|  |  | (*c*) Explain how missing data were addressed | 9 | “using three approaches to missing data: 1) complete case analysis (excluding participants eligible for sputum submission but for whom smear, Xpert MTB/Rif and/or culture data were missing); 2) multiple imputation of missing values for sex, age, HIV status, symptom status, X-ray status, sputum results, previous TB, TB contact, crowding and wealth variables, and 3) imputation of missing data for those eligible for sputum submission (cough or abnormal X-ray) with inverse probability weighting to represent all eligible individuals.” |
|  |  | (*d*) *Cohort study*—If applicable, explain how loss to follow-up was addressed  *Case-control study*—If applicable, explain how matching of cases and controls was addressed  *Cross-sectional study*—If applicable, describe analytical methods taking account of sampling strategy | 9 | “robust standard errors (calculated from observed between-cluster variability) to account for clustering…” |
|  |  | (*e*) Describe any sensitivity analyses | 9 | “Sensitivity analysis was also conducted for an alternate definition of a complete case, restricted to participants with available sputum result data and those who completed both screens.” |
| Results | | | | |
| Participants | 13* | (a) Report numbers of individuals at each stage of study—eg numbers potentially eligible, examined for eligibility, confirmed eligible, included in the study, completing follow-up, and analysed | 11 | “Between May 2019 and March 2020, 20,899 eligible adults were enumerated in 7,175 randomly selected and visited households… 76% (15,897) participated in the survey and underwent symptom screen; 13,490 (85%) had chest X-ray. 1,394/15,897 (9%) participants were eligible to submit sputum through reporting a cough of any duration and/or abnormal X-ray. Of these, 1,140 (82%) submitted at least one sputum sample and 900 submitted two sputum samples…” |
|  |  | (b) Give reasons for non-participation at each stage | 11 | Figure 1 |
|  |  | (c) Consider use of a flow diagram | 11 | Figure 1 |
| Descriptive data | 14* | (a) Give characteristics of study participants (eg demographic, clinical, social) and information on exposures and potential confounders | 11-12 | Table 1 + text |
|  |  | (b) Indicate number of participants with missing data for each variable of interest | 12 | “Of the 1,395 participants eligible to submit sputum 1,120 had valid smear results, 1,075 valid culture results, and 900 valid Xpert MTB/Rif results. 579 sputum-eligible participants were missing valid results from at least one sputum tests giving 15,318 complete cases (Table 1).” |
|  |  | (c) *Cohort study*—Summarise follow-up time (eg, average and total amount) | N/A | N/A |
| Outcome data | 15* | *Cohort study*—Report numbers of outcome events or summary measures over time | N/A | N/A |
|  |  | *Case-control study—*Report numbers in each exposure category, or summary measures of exposure | N/A | N/A |
|  |  | *Cross-sectional study—*Report numbers of outcome events or summary measures | 12-13 | “29 participants were identified with bacteriologically-confirmed TB … Of those 29, nine were smear-positive (all confirmed by Xpert MTB/Rif or culture) and the others Xpert/culture positive and smear negative (Figure 1 & Supplementary Table 1).” |
| Main results | 16 | (*a*) Give unadjusted estimates and, if applicable, confounder-adjusted estimates and their precision (eg, 95% confidence interval). Make clear which confounders were adjusted for and why they were included | 13 | “The overall prevalence of bacteriologically-confirmed TB was: 189 per 100,000 adults (95% CI 132-272) for the complete case model; 139 per 100,000 adults (95% CI: 71-272) for the multiple imputation model; and 150 per 100,000 adults (95% CI: 76-297) for the inverse probability weighted model (Figure 2).” |
|  |  | (*b*) Report category boundaries when continuous variables were categorized | 12 | Table 1 |
|  |  | (*c*) If relevant, consider translating estimates of relative risk into absolute risk for a meaningful time period | N/A | N/A |

Continued on next page

| Other analyses | 17 | Report other analyses done—eg analyses of subgroups and interactions, and sensitivity analyses | 13-16 | “Sensitivity analysis with the alternate complete case definition gave a TB prevalence of 223 per 100,000 adults (95% CI: 155-320)….” |
| --- | --- | --- | --- | --- |
| Discussion | | | | |
| Key results | 18 | Summarise key results with reference to study objectives | 17-20 | “The main finding from this survey is that the estimated adult prevalence of bacteriologically-confirmed pulmonary TB in Blantyre from this survey – 150 per 100,000 in the inverse probability weighted model – was more than 80% lower than the previous estimates for urban areas in the 2013-14 Malawi National TB Prevalence Survey…” |
| Limitations | 19 | Discuss limitations of the study, taking into account sources of potential bias or imprecision. Discuss both direction and magnitude of any potential bias | 20-21 | “This study has some limitations, including low precision from the small number of cases in our survey, due to lower than anticipated prevalence, and low rates of participation, particularly amongst working age men….” |
| Interpretation | 20 | Give a cautious overall interpretation of results considering objectives, limitations, multiplicity of analyses, results from similar studies, and other relevant evidence | 17-21 | “…Our study demonstrates a substantial decrease in TB prevalence in urban Malawi over the eight years before the COVID-19 pandemic….” |
| Generalisability | 21 | Discuss the generalisability (external validity) of the study results | 21 | “…future case-finding in Blantyre and similar urban centres in sub-Saharan Africa, should target the highest risk groups such as working-age and older men…” |
| Other information | |  | | |
| Funding | 22 | Give the source of funding and the role of the funders for the present study and, if applicable, for the original study on which the present article is based | N/A | Not in manuscript (as requested) but included in submission |

*Give information separately for cases and controls in case-control studies and, if applicable, for exposed and unexposed groups in cohort and cross-sectional studies.

**Note:** An Explanation and Elaboration article discusses each checklist item and gives methodological background and published examples of transparent reporting. The STROBE checklist is best used in conjunction with this article (freely available on the Web sites of PLoS Medicine at http://www.plosmedicine.org/, Annals of Internal Medicine at http://www.annals.org/, and Epidemiology at http://www.epidem.com/). Information on the STROBE Initiative is available at www.strobe-statement.org.
