# Supplementary material for: Prevalence of bacteriologically-confirmed pulmonary tuberculosis in urban Blantyre, Malawi 2019–20: Substantial decline compared to 2013–14 national survey
Source: PLOS Glob Public Health. 2023 Oct 20;3(10):e0001911. doi: 10.1371/journal.pgph.0001911 (PMC10588852; doi:10.1371/journal.pgph.0001911)
Supplement: S1 Text — including: Table A: Clinical and microbiological characteristics of confirmed TB casesTable B: Participants diagnosed with TB disaggregated by screening category and HIV statusTable C: Prevalence of smear-positive TB disease per 100,000 adults with robust standard errors used to calculate 95% confidence intervals, from complete case and inverse probability weighting analysisTable D: Characteristics of complete case participantsTable E: Prevalence of TB disease per 100,000 adults with robust standard errors used to calculate 95% confidence intervals, using WHO-recommended expanded age and HIV groupsTable F: Risk factors for prevalent bacteriologically-confirmed TB using WHO recommended, expanded age and HIV categories with robust standard errors used to calculate 95% confidence intervals (DOCX) [file pgph.0001911.s002.docx]

**S1 Text: Supplementary Material**

**Table A: Clinical and microbiological characteristics of confirmed TB cases**

| **Characteristics** | | | | **Symptom screening** | | | | | | **X-ray** | **Sputum results** | | | |
| --- | --- | --- | --- | --- | --- | --- | --- | --- | --- | --- | --- | --- | --- | --- |
| **Sex** | **Age** | **HIV status** | **Previous TB?** | **Cough** | **Chronic cough** | **Night sweats** | **Weight loss** | **Fever** | **Any TB symptoms** | **Chest X-ray** | **Smear** | **Xpert** | **Culture result** | **Culture ID** |
| Male | 45 | HIV negative | No | Yes | Yes | Yes | Yes | Yes | Yes | Abnormal | Negative | Positive | Negative | ND |
| Female | 32 | HIV positive | No | Yes | No | No | No | No | Yes | Normal | Negative | Negative | Positive | MTB |
| Female | 34 | HIV positive ART | No | Yes | Yes | Yes | Yes | No | Yes | Abnormal | Negative | Positive | Positive | MTB |
| Female | 29 | HIV negative | No | Yes | Yes | No | No | No | Yes | *-* | Negative | Positive | Positive | MTB |
| Male | 25 | HIV negative | No | Yes | Yes | No | No | No | Yes | Abnormal | Negative | Positive | Positive | MTB |
| Female | 19 | HIV negative | No | Yes | Yes | No | No | No | Yes | Abnormal | Positive | Positive | Positive | MTB |
| Female | 26 | HIV negative | No | Yes | Yes | No | No | Yes | Yes | Abnormal | Positive | Positive | Positive | MTB |
| Female | 22 | HIV negative | No | Yes | No | No | No | No | Yes | Normal | Negative | Negative | Positive | MTB |
| Male | 33 | HIV negative | Yes | Yes | No | Yes | No | Yes | Yes | Abnormal | Positive | Positive | Positive | MTB |
| Male | 19 | HIV negative | No | Yes | No | No | No | No | Yes | Abnormal | Negative | Negative | Positive | MTB |
| Male | 56 | HIV positive ART | No | Yes | Yes | No | No | Yes | Yes | Normal | Positive | Positive | Positive | MTB |
| Male | 45 | HIV positive ART | No | Yes | Yes | No | No | No | Yes | Abnormal | Negative | Positive | Negative | ND |
| Male | 40 | HIV negative | Yes | Yes | Yes | No | No | No | Yes | Normal | Positive | Positive | Positive | MTB |
| Female | 61 | HIV negative | No | No | No | No | No | No | No | Abnormal | Negative | Positive | Positive | MTB |
| Female | 89 | HIV negative | No | No | No | No | No | No | No | Abnormal | Negative | Positive | Negative | ND |
| Male | 27 | HIV negative | Yes | No | No | No | No | Yes | Yes | Abnormal | Negative | Positive | Contaminated | ND |
| Male | 27 | HIV negative | No | No | No | No | No | No | No | Abnormal | Negative | Negative | Positive | MTB |
| Male | 33 | HIV negative | Yes | No | No | No | No | No | No | Abnormal | Negative | Negative | Positive | MTB |
| Male | 43 | HIV negative | No | No | No | No | No | Yes | Yes | Abnormal | Negative | Positive | Positive | MTB |
| Male | 30 | HIV negative | No | No | No | Yes | No | No | Yes | Abnormal | Positive | Positive | Positive | MTB |
| Female | 38 | HIV negative | No | No | No | No | No | No | No | Abnormal | Negative | Negative | Positive | MTB |
| Male | 30 | HIV negative | No | No | No | Yes | No | Yes | Yes | Abnormal | Negative | Positive | Negative | ND |
| Female | 19 | HIV negative | No | No | No | No | No | No | No | *-* | Negative | Negative | Positive | MTB |
| Male | 44 | HIV negative | No | No | No | No | No | No | No | Normal | Positive | Positive | Positive | MTB |
| Male | 36 | HIV negative | No | No | No | Yes | No | No | Yes | Normal | Negative | Positive | Negative | ND |
| Male | 54 | HIV negative | No | No | No | No | No | No | No | Abnormal | Negative | Negative | Positive | MTB |
| Female | 37 | HIV positive ART | No | No | No | No | No | No | No | Abnormal | Negative | Positive | Positive | MTB |
| Male | 22 | HIV negative | No | No | No | No | No | No | No | Abnormal | Positive | Positive | Negative | ND |

**Table B: Participants diagnosed with TB disaggregated by screening category and HIV status**

|  | | | **Participants diagnosed with TB** | | |
| --- | --- | --- | --- | --- | --- |
| **Screening category** | **Screen positive participants** | **Participants diagnosed with TB (%)** | **HIV-negative** | **HIV-positive  on ART** | **HIV-positive  (no ART)** |
| Symptom (cough) only | 747 | 5* (17%) | 3 | 1 | 1 |
| Abnormal CXR only | 406 | 12 (41%) | 11 | 1 | - |
| Symptom and abnormal CXR | 241 | 9 (31%) | 6 | 3 | - |
| No symptom / CXR normal or not recorded | 84 | 3 (10%) | 3 | - | - |
| Total | 1,478 | 29 | 23 | 5 | 1 |

CXR: Chest X-ray

*One person identified with TB had cough recorded but no CXR record

**Table C: Prevalence of smear-positive TB disease per 100,000 adults with robust standard errors used to calculate 95% confidence intervals, from complete case and inverse probability weighting analysis**

|  | Total (n) | Smear+ TB (n) |  | Prevalence (95% CI) | |
| --- | --- | --- | --- | --- | --- |
|  |  |  |  | Complete case | Inverse weighting |
| *All participants* | 15318 | 9 |  | 69 (36-133) | 37 (9-169) |
| *Sex* |  |  |  |  |  |
| Female | 9423 | 2 |  | 25 (6-102) | 18 (4-71) |
| Male | 5895 | 9 |  | 136 (28-651) | 84 (18-400) |
| *Age* |  |  |  |  |  |
| 18-24 | 5811 | 2 |  | 40 (10-159) | 28 (7-112 |
| 25-49 | 4356 | 5 |  | 77 (15-398) | 48 (9-250) |
| 50+ | 582 | 2 |  | 130 (18-915) | 141 (23-852) |
| *HIV status* |  |  |  |  |  |
| HIV negative | 15318 | 7 |  | 62 (30-130) | 42 (20-88) |
| HIV positive | 5895 | 2 |  | 115(24-550) | 132 (33-520) |
| *Previous diagnosis* |  |  |  |  |  |
| No previous TB | 14895 | 25 |  | 55 (26-116) | 42 (21-86 |
| Previous TB | 423 | 4 |  | 543 (113-2580) | 349 (74-1640) |

**Table D: Characteristics of complete case participants**

|  | Total (n) | TB (n) |
| --- | --- | --- |
|  |  |  |
| *All participants* | 15318 | 29 |
| *Sex* |  |  |
| Female | 9423 | 11 |
| Male | 5895 | 18 |
| *Age* |  |  |
| 18-24 | 5811 | 5 |
| 25-49 | 7723 | 19 |
| 50+ | 1779 | 5 |
| *HIV status* |  |  |
| HIV negative | 12815 | 23 |
| HIV positive | 1877 | 6 |
| *Previous diagnosis* |  |  |
| No previous TB | 14895 | 25 |
| Previous TB | 423 | 4 |
| *TB contact (within 12 months)* |  |  |
| No | 14608 | 27 |
| Yes | 681 | 2 |
| *Crowding, persons per room* |  |  |
| <1 | 7374 | 11 |
| 1-2 | 7139 | 16 |
| >2 | 672 | 2 |
| *Wealth quartile** |  |  |
| 1 | 3398 | 7 |
| 2 | 3686 | 5 |
| 3 | 3918 | 10 |
| 4 | 4183 | 7 |

* Probability of being below the poverty line from 1 (most likely) to 4 (least likely)

104 participants had no crowding or wealth quartile data recorded

**Table E: Prevalence of TB disease per 100,000 adults with robust standard errors used to calculate 95% confidence intervals, using WHO-recommended expanded age and HIV groups**

|  | Total (n) | TB (n) |  | Prevalence (95% CI) | | |
| --- | --- | --- | --- | --- | --- | --- |
|  |  |  |  | Complete case | Fully imputed | Inverse weighting |
| *All participants* | 15318 | 29 |  | 189 (132-272) | 139 (71-272) | 159 (78-324) |
| *Sex* |  |  |  |  |  |  |
| Female | 9423 | 11 |  | 117 (65-211) | 97 (54-176) | 97 (53-178) |
| Male | 5895 | 18 |  | 305 (144-645) | 198 (94-415) | 259 (115-580) |
| *Age* |  |  |  |  |  |  |
| 18-24 | 5811 | 5 |  | 86 (36-207) | 67 (29-165) | 65 (27-158) |
| 25-34 | 4356 | 11 |  | 253 (88-724) | 184 (64-528) | 220 (75-645) |
| 35-44 | 2649 | 6 |  | 227 (69-739) | 149 (46-489) | 160 (48-537) |
| 45-54 | 1205 | 4 |  | 332 (89-1228) | 274 (79-940) | 495 (119-2035) |
| 55-64 | 710 | 2 |  | 282 (55-1440) | 201 (40-1027) | 195 (37-1032) |
| 65+ | 582 | 1 |  | 172 (20-1459) | 126 (15-1069) | 124 (14-1055) |
| *HIV status* |  |  |  |  |  |  |
| HIV negative | 15318 | 23 |  | 179 (119-270) | 132 (89-199) | 177 (106-293) |
| HIV positive ART | 9423 | 1 |  | 303 (115-795) | 263 (107-643) | 277 (105-731) |
| HIV positive | 5895 | 5 |  | 439 (59-3198) | 325 (44-2362) | 443 (59-3272) |
| *Previous diagnosis* |  |  |  |  |  |  |
| No previous TB | 14895 | 25 |  | 168 (113-248) | 128 (87-189) | 164 (102-264) |
| Previous TB | 423 | 4 |  | 946 (329-2687) | 613 (214-1742) | 702 (233-2096) |

Notes:

HIV status as identified through testing in prevalence survey, or if no test as reported in individual survey

5 of complete cases no age recorded (3 HIV negative, 1 HIV positive & 1 HIV unknown)

626 participants with HIV unknown status but no TB cases amongst them

Previous TB includes one currently on TB treatment

**Table F: Risk factors for prevalent bacteriologically-confirmed TB using WHO recommended, expanded age and HIV categories with robust standard errors used to calculate 95% confidence intervals**

|  |  | Multivariate analysis | | |
| --- | --- | --- | --- | --- |
| Variable | Univariate OR | Complete case OR (95% CI) | Fully imputed OR (95% CI) | Inverse weighting OR (95% CI) |
| *Sex* |  |  |  |  |
| Female | 1 | 1 | 1 | 1 |
| Male | *2.62 (1.24-5.55)* | *2.76 (1.28-5.95)* | *2.06 (0.97-4.41)* | *2.38 (1.14-4.94)* |
| *Age, years* |  |  |  |  |
| 18-24 | 1 | 1 | 1 | 1 |
| 25-34 | 2.94 (1.02-8.47) | 3.16 (1.09-9.15) | 2.65 (0.86-8.22) | 3.34 (1.09-10.27) |
| 35-44 | 2.64 (0.80-8.65) | 2.65 (0.76-9.20) | 1.92 (0.55-6.78) | 2.21 (0.60-8.18) |
| 45-54 | 3.87 (1.04-14.43) | 3.26 (0.83-12.80) | 3.00 (1.05-8.62) | 5.85 (1.24-27.49) |
| 55-64 | 3.28 (0.63-16.97) | 2.80 (0.53-14.77) | 2.29 (0.42-12.40) | 2.27 (0.43-12.14) |
| 65+ | 2.00 (0.23-17.19) | 1.83 (0.21-15.82) | 1.69 (0.19-14.88) | 1.81 (0.20-16.23) |
| *HIV/ART status* |  |  |  |  |
| HIV- | 1 | 1 | 1 | 1 |
| HIV+ on ART | 1.69 (0.64-4.46) | 1.10 (0.30-4.07) | 1.49 (0.55-4.03) | 1.10 (0.37-3.24) |
| HIV+ not on ART | 2.45 (0.33-18.38) | 1.95 (0.22-17.09) | 2.20 (0.27-18.09) | 1.96 (0.21-18.14) |
| HIV unknown | 0 | 0 | 0 | 0 |
| *Previous TB* | 5.68 (1.96-16.42) | 4.25 (0.87-20.67) | 3.41 (0.97-12.01) | 3.10 (0.83-11.56) |
| *TB contact (within 12 months)* | 1.59 (0.38-6.71) | 1.33 (0.30-5.94) | 1.29 (0.29-5.83) | 1.23 (0.25-6.04) |
| *Crowding, persons per room* |  |  |  |  |
| <1 | 1 | 1 | 1 | 1 |
| 1-2 | 1.50 (0.70-3.24) | 1.65 (0.73-3.72) | 1.67 (0.83-3.36) | 2.16 (0.95-4.92) |
| >2 | 2.00 (0.44-9.04) | 2.46 (0.44-13.58) | 2.57 (0.48-13.60) | 1.91 (0.37-9.96) |
| *Wealth quartile* |  |  |  |  |
| 1 | 1 | 1 | 1 | 1 |
| 2 | 0.66 (0.21-2.08) | 0.64 (0.20-2.05) | 0.58 (0.17-1.94) | 0.59 (0.16-2.12) |
| 3 | 1.24 (0.47-3.26) | 1.10 (0.39-3.11) | 1.04 (0.37-2.92) | 1.26 (0.42-3.79) |
| 4 (top) | 0.81 (0.28-2.32) | 0.67 (0.20-2.23) | 0.63 (0.19-2.07) | 0.70 (0.22-2.23) |
